# Simple HPLC-UV Method for Therapeutic Drug Monitoring of 12 Antiepileptic Drugs and Their Main Metabolites in Human Plasma

**DOI:** 10.3390/molecules28237830

**Published:** 2023-11-28

**Authors:** Daniela Milosheska, Robert Roškar

**Affiliations:** University of Ljubljana, Faculty of Pharmacy, Aškerčeva Cesta 7, 1000 Ljubljana, Slovenia

**Keywords:** antiepileptic drugs, human plasma, therapeutic drug monitoring, bioanalytical method validation, high-performance liquid chromatography

## Abstract

The objective of the present report was to develop and validate a simple, selective, and reproducible high-performance liquid chromatography method with UV detection suitable for routine therapeutic drug monitoring of the most commonly used antiepileptic drugs and some of their metabolites. Simple precipitation of plasma proteins with acetonitrile was used for sample preparation. 10,11-dihydrocarbamazepine was used as an internal standard. Chromatographic separation of the analytes was achieved by gradient elution on a Phenyl–Hexyl column at 40 °C, using methanol and potassium phosphate buffer (25 mM; pH 5.1) as a mobile phase. The method was validated according to the FDA guidelines for bioanalytical method validation. It showed to be selective, accurate, precise, and linear over the concentration ranges of 1–50 mg/L for phenobarbital, phenytoin, levetiracetam, rufinamide, zonisamide, and lacosamide; 0.5–50 mg/L for lamotrigine, primidone, carbamazepine and 10-monohydroxycarbazepine; 0.2–10 mg/L for carbamazepine metabolites: 10,11-trans-dihydroxy-10,11-dihydrocarbamazepine and carbamazepine-10,11-epoxide; 0.1–10 mg/L for oxcarbazepine; 2–100 mg/L for felbamate and 3–150 mg/L for ethosuximide. The suitability of the validated method for routine therapeutic drug monitoring was confirmed by quantification of the analytes in plasma samples from patients with epilepsy on combination antiepileptic therapy.

## 1. Introduction

Antiepileptic drugs (AEDs) are used to decrease the frequency and/or severity of seizures in people with epilepsy. AEDs are classified into three generations and are characterized by extensive pharmacological and structural diversity [1]. Therapeutic drug monitoring (TDM) as a clinical specialty used for optimization and individualization of drug therapy in the general and special populations is initiated for a number of AEDs, as most of them show pronounced variability in pharmacokinetics [2]. TDM of first-generation AEDs, including phenytoin (PHE), phenobarbital (PHB), primidone (PRM), ethosuximide (ETH), carbamazepine (CBZ), and valproic acid is already a common practice for dosage adjustment because of their narrow therapeutic window with toxicity and neurological side effects as common problems [1,2]. Also, there is growing evidence about the usefulness of TDM of the newer generations of AEDs such as lamotrigine (LTG), oxcarbazepine (OXC), rufinamide (RUF), vigabatrin, felbamate (FEL), stiripentol, topiramate, pregabalin, levetiracetam (LEV), gabapentin, tiagabine, zonisamide (ZON), lacosamide (LAC), eslicarbazepine acetate, retigabine, and perampanel. However, they are characterized by more predictable pharmacokinetics and lack of documented concentration-effect correlation [2,3]. Considering the large pharmacokinetic interindividual variability of all AEDs, TDM as a valuable tool for therapy individualization contributes to quality assurance of AED treatment, assessment of drug compliance, management of uncontrolled seizures, prevention and management of drug–drug interactions, overdoses, and clinical toxicity [1,2]. TDM is particularly indicated for AED treatment optimization in special populations such as children, pregnant women, and the elderly [2].

Because TDM aims to optimize patient outcomes by guiding the drug regimen based on information about drug concentrations in body fluids (usually serum or plasma), precise and accurate laboratory measurement of drug concentrations is critical for successful TDM. The proposed “individual therapeutic concentration” approach is more flexible compared to fixed reference ranges and can be applied to any drug, including those for which no clear reference ranges are reported [2,4]. Therefore, the availability of suitable analytical methods is necessary to support studies designed to provide evidence for the usefulness of the routine monitoring of AEDs.

To date, several methods based on high-performance liquid chromatography (HPLC) coupled to ultraviolet or mass spectrometry detection for the simultaneous quantification of AEDs in human plasma or serum have been reported and reviewed elsewhere [5,6,7,8]. Some of the methods also include quantification of the main metabolites, such as 10-monohydroxycarbazepine (MHD) and carbamazepine-10,11-epoxide (CBZ-E), pharmacologically active metabolites of OXC and CBZ, respectively [9,10,11,12,13,14,15].

MHD is responsible for the anticonvulsant activity of OXC as the majority of the administered drug is rapidly and extensively metabolized to MHD [16]. Therefore, the reported reference range of therapeutic concentrations of OXC refers to the MHD since the parent drug OXC is only 10% present in the body fluids [17]. On the other hand, CBZ-E is not routinely monitored and included in the targeted therapeutic range for CBZ, although it contributes significantly to the therapeutic effect with concentrations of approximately 15 to 20% of the total carbamazepine concentration at steady state [18]. Moreover, CBZ-E is responsible for most of the undesirable side effects and toxicity of CBZ [19]. This metabolite is then almost completely converted to the inactive 10,11-trans-dihydroxy-10,11-dihydrocarbamazepine (DIOL) [20]. Minor amounts of MHD are also oxidized to DIOL [21]. Another AED that is mainly metabolized to its active metabolite is PRM, which is converted to PHB in the liver [22]. The development of analytical methods able to simultaneously quantify different AEDs is warranted because polytherapy is often used in the treatment of epilepsy, and TDM can be particularly useful during switching therapies and evaluating pharmacokinetic drug–drug interactions. However, most published methods for simultaneous analysis of multiple AEDs (>10) and their metabolites use complex mass detection that is not cost-effective for routine use [12,15,23] or multiple sample preparation and HPLC-UV analytical conditions for different groups of AEDs [24]. Other published HPLC-UV methods include fewer analytes in a single run: four [25], five [26], six [9,11,27], nine [10,28], and ten [29].

**Figure 1:**
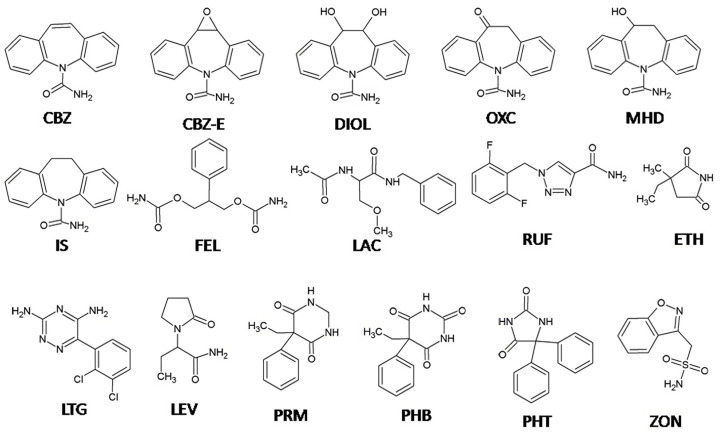
Chemical structures of the tested AEDs and the internal standard (IS).

The aim of our study was to develop a simple and sensitive analytical method for TDM of the most commonly used old and new AEDs suitable for direct UV detection including CBZ, PHB, PHE, PRM, FEL, ETH, OXC, ZON, RUF, LTG, LEV and LAC, as well as the OXC and CBZ metabolites MHD, CBZ-E and DIOL (Figure 1). To our knowledge, this is the first HPLC-UV method reported for simultaneous analysis of the selected 12 AEDs and their three metabolites (MHD, CBZ-E, and DIOL) in a single run using only 100 µL of human plasma. The suitability of the method for routine TDM was confirmed by analysis of plasma samples from patients with epilepsy undergoing combination AED therapy.

## 2. Results and Discussion

### 2.1. Optimization of the Analytical Method

**Figure 2:**
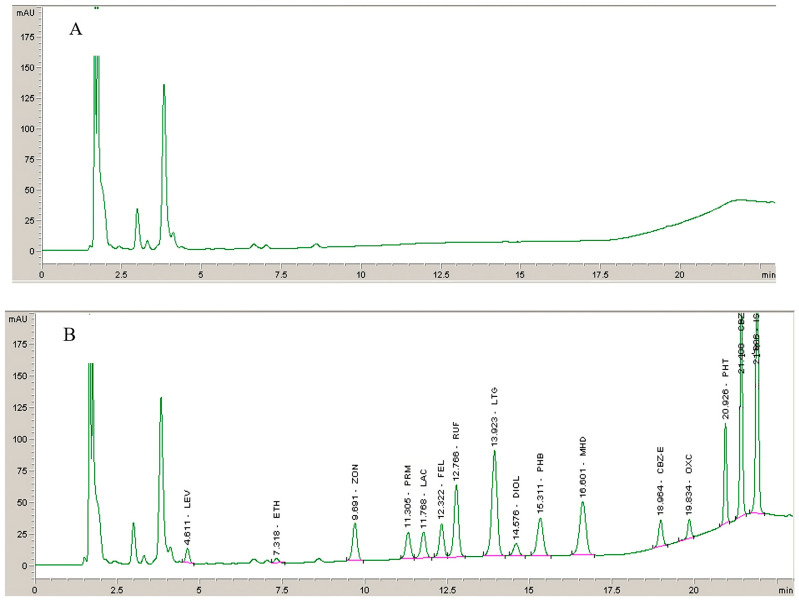
Chromatograms obtained from (**A**) deproteinized blank plasma and (**B**) deproteinized plasma sample spiked with AEDs and their metabolites at QCL level and IS (10 mg/L).

Our aim was to develop and validate a simple, sensitive, and selective analytical method for the simultaneous determination of the most commonly used AEDs and some of their metabolites, suitable for routine TDM. The AEDs tested were selected considering their physicochemical properties and their suitability for direct UV detection. AEDs such as topiramate, vigabatrin, valproic acid, and pregabalin, which require derivatization with fluorescent agents or mass spectrometry for their detection, were not considered potential analytes in our study [30,31,32,33]. During method development, chromatographic conditions were optimized to achieve the best separation of all 15 analytes, including internal standard (IS). First, several reversed-phase analytical columns (C8, C18, and Phenyl–Hexyl), isocratic and gradient elution with different mobile phase compositions (mixtures of methanol or acetonitrile with phosphate buffer at different ratios and pH values), injection volumes (5–20 µL), and column temperatures (35–50 °C) were evaluated. Since the majority of the tested AEDs contain aromatic rings in their structure (Figure 1), we chose the Phenyl–Hexyl column as an alternative to the other reversed-phase columns with complementary selectivity, which is recommended for compounds with aromatic rings. The Phenyl-Hexyl column has unique selectivity in a challenging chromatographic separation of active ingredients with similar structures because of the retention mechanism based on interactions between the various analyte molecules and the phenyl-bonded phase [34]. Consequently, methanol was used as part of the mobile phase because, compared to acetonitrile, it enhances the π–π interactions between the analytes’ aromatic ring and the phenyl stationary phase and provides increased retention and additional selectivity. During optimization, we found that the separation of PHB was significantly dependent on the pH of the buffer and column temperature. Due to its weakly acidic character (pKa = 7.1) [35], we selected an acidic aqueous mobile phase (two units below pKa) in which PHB was present in the unionized form. The chromatographic separation of the 16 selected analytes was challenging due to the different physicochemical properties, mainly because of the log P between −0.8 (LEV) and 2.9 (CBZ) [35] and the number of aromatic rings (up to three, Figure 1), which required gradient elution. Different starting point compositions of mobile phase A and B and gradient programs focusing on the separation of analytes with very similar chromatographic behavior (PRM-RUF, Figure 2) were tested by applying a very shallow gradient in this range.

Optimal separation for all analytes tested was achieved on the Luna Phenyl–Hexyl column by gradient elution using a mixture of potassium phosphate buffer (25 mM, pH 5.1) and methanol as the mobile phase with a flow rate of 1 mL/min at 210 nm. To the best of our knowledge, this is the first HPLC-UV method using a Phenyl–Hexyl column to separate AEDs, as other published methods use C18 columns and acetonitrile as an organic modifier [5,6,7]. The instrumentation and chromatographic conditions are described in detail in Section 3.2. During method development, chromatograms were recorded simultaneously at three different wavelengths (210, 235, and 280 nm) using the DAD detector, considering the absorbance maximum of the analyzed compounds. Since we were guided by the simplicity of the method, 210 nm was chosen as the optimal wavelength because it provided the required sensitivity for the quantification of the compounds at low concentrations without interference.

Chloramphenicol, guanabenz, and 10,11-dihydrocarbamazepine were tested as internal standards. We chose 10,11-dihydrocarbamazepine as the IS because it is structurally related to some of the tested analytes and achieved suitable retention time and resolution relative to other analytes. The results obtained during the validation process confirmed its suitability.

Our goal was to develop a simple and selective method for routine TDM of AEDs, so we chose protein precipitation with an organic solvent as the sample preparation method. Protein precipitation was chosen as the first step of sample preparation based on previous studies that have also demonstrated the suitability of the procedure for the extraction of multiple AEDs from plasma [15]. To achieve optimal AED extraction from a small plasma volume (100 µL), different precipitation solvents were tested: methanol, acetonitrile, and methanol/acetonitrile mixture (50/50, *v*/*v*) at room temperature and ice-cold. Protein precipitation was further optimized by testing different volumes of precipitation solvent (300, 500, 800 µL) and mixing times (30 and 60 s). In the second step, different volumes of supernatant (250, 450, 700, 750, and 800 µL—considering the volume of the precipitation solvent) were evaporated to dryness under a stream of nitrogen at 40 °C and dissolved in methanol/water as reconstitution solvent (50/50, *v*/*v*) with 1 min of vortexing. There were minor differences in recoveries between tested conditions (within 15%); however, better reproducibility was achieved with higher precipitation volumes. Good selectivity without peaks of endogenous substances at the retention times of the tested AEDs and their metabolites, as well as reproducible and high recoveries, were obtained using only 100 µL of plasma and 800 µL of ice-cold acetonitrile. The optimal procedure is described in detail in the sample preparation section. The optimized sample preparation procedure resulted in clean samples suitable for the intended analysis. 

To our knowledge, this is the first HPLC-UV method for simultaneous analysis of the selected AEDs in a single run requiring only 100 µL of plasma, making it suitable for TDM in sensitive populations such as children and the elderly. Compared to the other HPLC-UV analytical methods for the analysis of one or a selected group of AEDs, our method enables cost-effective simultaneous quantification of 12 most commonly used AEDs and 3 metabolites, with single sample preparation in a single run. Other published HPLC-UV methods for multiple AEDs include fewer analytes in a single run; use higher plasma volumes: 200 µL [24], 250 µL [26], 500 µL [9,10], 1 mL [25]; more complex solid-phase extraction [10,28], microextraction [11] or have comparable analytical run time but with fewer analytes included [24,25,26,27,28]. In addition, the method is suitable for routine analysis in settings without expensive equipment such as LC-MS because it can be performed even on an HPLC with a single variable-wavelength detector. Also, the developed method has a wider analytical range than the proposed therapeutic range, compared with other HPLC methods for some analytes covering lower concentrations [24,26,27,29] or higher concentrations [9,11,26,27,28,29], supporting the suitability of the method for routine TDM.

### 2.2. Method Validation

#### 2.2.1. Selectivity

No endogenous interferences at the retention times of the analytes and IS were detected. A representative chromatogram of a blank plasma sample and a chromatogram at the LLOQ concentration level are shown in Figure 2. Analysis of plasma samples from patients with epilepsy treated in combination with other AEDs such as topiramate, vigabatrin, valproic acid, clobazam, clonazepam, and pregabalin showed no interfering peaks. Additionally were tested few non-AEDs (acetaminophen, diclofenac, omeprazole, fluoxetine, alprazolam, ketoprofen, ibuprofen, diazepam, sertraline, enalapril, ranitidine, oxazepam, lorazepam, quetiapine, bromazepam, escitalopram, and acetylsalicylic acid) that could potentially be coadministered. None of the tested compounds showed interfering peaks at the retention time of the analytes included in our method. 

#### 2.2.2. Linearity

The linearity of the method was demonstrated within the defined analytical range for all tested AEDs and metabolites. The regression equations of the calibration curves and corresponding determination coefficients (R^2^) obtained by non-weighted linear regression analysis are presented in Table 1. The proposed analytical ranges are wider than the defined therapeutic ranges for each AED, covering concentrations above and below this range that support the suitability of the method for routine TDM.

#### 2.2.3. Accuracy and Precision

Results for inter-day and intra-day accuracy and precision are presented in Table 2. The intra- and inter-day precisions, expressed as CV, were below 11%. Bias values for intra- and inter-day accuracies were within ±6.76% for all tested analytes, which is in accordance with the defined acceptance criteria.

#### 2.2.4. Recovery and Stability

The results of the mean recoveries for each analyte are presented in Table 3. The mean recovery for IS was 100.2 ± 1.6%, confirming the suitability of the selected IS. We achieved high recoveries with absolute mean values ranging from 93.4 to 102.1% for all analytes except for OXC, which achieved 83.9%. Overall, recovery results demonstrated that the sample preparation method of the plasma samples is precise, consistent, and reproducible.

The stability data of the analytes for different experimental conditions are summarized in Table 3. No significant loss was observed during the stability testing period since all obtained results were in agreement with the stated acceptance criteria (±15%). Although the IS was used for response normalization and assessed daily by reviewing the results of the analytical run, the confirmed stability of the tested analytes indirectly confirmed the stability of the IS, since the selected IS is a close chemical analog to some of the analyzed compounds stored under identical conditions [36].

### 2.3. Clinical Application of the Method

**Figure 3:**
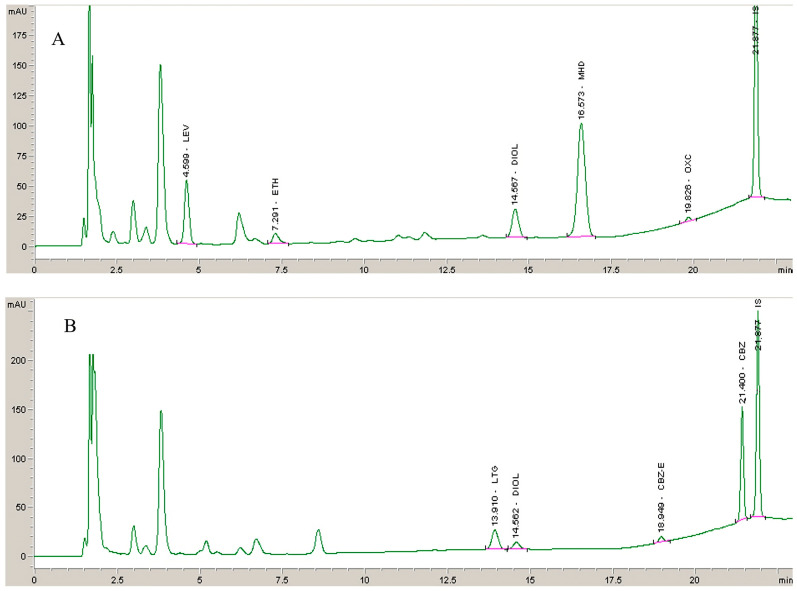
Representative chromatogram of plasma sample obtained from patients with epilepsy (**A**) treated with OXC (450 mg), ETH (475 mg), and LEV (1050 mg) and (**B**) treated with LTG (300 mg) and CBZ (800 mg).

The suitability and applicability of the method for routine TDM of the analyzed AEDs were confirmed by analyzing plasma samples from 13 patients with epilepsy (Table 4). Representative chromatograms of plasma samples obtained from two patients treated with OXC (450 mg), ETH (475 mg), and LEV (1050 mg) (ID 1) and LTG (300 mg) and CBZ (800 mg) (ID13) are presented in Figure 3. 

As shown, the peak shape and resolution of the analytes from real samples are similar to those obtained using spiked plasma samples without interferences. Also, OXC and CBZ metabolites were successfully quantified. The large variability in the pharmacokinetics of AEDs and the possibility of drug-drug interactions due to concomitant use of enzyme inducers (such as phenytoin, phenobarbital, primidone, carbamazepine, oxcarbamazepine) and inhibitors (such as valproic acid, felbamate, rufinamide) are some of the reasons for routine TDM. For example, with LTG, large variability is observed in samples of patients receiving different daily doses (from 150 mg to 600 mg). Because lamotrigine is intensively metabolized by glucuronidation, an induction/inhibition effect may be attributed to concomitantly administered antiepileptic drugs. The highest measured plasma concentrations are observed with concomitant administration of valproic acid (enzyme inhibitor) in ID 8 or lacosamide (non-enzymatic AED) in ID 9. In contrast, LTG concentrations are lower with administration of enzyme inducers (CBZ, PHB), and some results are below the reported reference therapeutic range (ID 5, 10, 12, 13; Table 4).

Simultaneous measurement of AEDs and their metabolites allows more comprehensive TDM and, in the case of OXC, CBZ, and PRM, determination of the metabolite-to-parent ratio, which can be used to identify individuals with different metabolic clearance, as these values reflect the enzymatic activities involved in the metabolism of the respective drugs and provide valuable information on metabolizer status (e.g., ultrarapid or poor metabolizer), medical compliance, and drug–drug interactions at the pharmacokinetic level [37].

In patients receiving carbamazepine, the calculated metabolite-to-parent ratio was consistent with the reported range in the literature (0.07–0.25) [37] when considering CBZ-E concentration as the major active metabolite. For OXC, on the other hand, there is no reported range, but it can be easily calculated from the measured concentrations of MHD and OXC from Table 4 (7.3–44). When monitoring OXC and CBZ concentrations, it is useful to monitor DIOL concentrations in addition to the concentrations of the major active metabolite. However, it does not play a clinically relevant role from a pharmacological point of view but provides a deeper insight into individual metabolism. From the results presented in Table 4, it is evident that there is a large interindividual variability, which supports the idea of routine TDM of AEDs using such methods with wider analytical ranges and the possibility of metabolite measurements. 

## 3. Materials and Methods

### 3.1. Chemicals and Reagents

CBZ, CBZ-E, PHB, PHE, PRM, FEL, ETH, and 10,11-dihydrocarbamazepine (IS) were purchased from Sigma Aldrich (Steinheim, Germany). OXC, MHD, ZON, LTG, LAC, and LEV were purchased from Sequoia Research (Pangbourne, UK). DIOL and RUF were obtained from Santa Cruz Biotechnology (Paso Robles, CA, USA). Methanol and acetonitrile, both of HPLC grade, were purchased from Sigma Aldrich (Steinheim, Germany). Used potassium dihydrogen phosphate and sodium hydroxide were obtained from Merck (Darmstadt, Germany). Ultrapure water was obtained by an A10 Advantage Milli-Q water purification system (Millipore Corp., Billerica, MA, USA).

### 3.2. Instrumentation and Chromatographic Conditions

The chromatographic analysis was carried out on an Agilent 1100 series HPLC system (Waldbronn, Germany) equipped with a vacuum degasser, binary pump, autosampler, column thermostat, UV-DAD detector, and ChemStation software (B02.01). Several chromatographic columns were tested: Zorbax C8 and Zorbax Eclipse Plus C18 (150 × 4.6 mm, 5 μm; Agilent Technologies, Santa Clara, CA, USA), Gemini C18, Luna C18 and Phenyl–Hexyl (150 × 4.6 mm, 5 μm; Phenomenex, Torrance, CA, USA). Separation was performed on a Luna Phenyl–Hexyl column (150 × 4.6 mm, 5 μm; Phenomenex, Torrance, CA, USA) coupled to the Gemini C6-Phenyl, (4.0 × 3.0 mm) guard precolumn (Phenomenex, Torrance, CA, USA), using gradient elution with potassium phosphate buffer (25 mM; pH 5.1) and methanol as a mobile phase (Table 5) at flow rate of 1.0 mL/min. The total run time was 27 min. The mobile phase A was filtered through a 0.45 μm filter under vacuum and ultrasonically degassed before analysis. The column temperature was 40 °C, and the autosampler temperature was kept at 10 °C. A sample volume of 20 μL was injected, and the detection of the analytes was performed at 210 nm.

### 3.3. Preparation of Solutions

Stock solutions of CBZ (10.0 mg/mL), CBZ-E (2.5 mg/mL), PHB (10.0 mg/mL), PHT (10.0 mg/mL), PRM (10.0 mg/mL), FEL (3,0 mg/mL), ETH (10.0 mg/mL), OXC (2.5 mg/mL), MHD (5.0 mg/mL), ZON (2.5 mg/mL), LTG (2.5 mg/mL), LAC (2.5 mg/mL), LEV (2.5 mg/mL), DIOL (2.0 mg/mL), RUF (2.5 mg/mL) and IS (2.5 mg/mL) were prepared by dissolving appropriate amounts of each compound in methanol. The stock solution of IS was diluted with methanol:water (50:50, *v*/*v*) to prepare the working solution (50 mg/L) used for the preparation of the calibration standard and quality control standard solutions. Calibration standard solutions containing all analytes and IS were prepared fresh daily from stock solutions by further dilution to obtain final plasma concentrations of: 1, 2.5, 5, 7.5, 10, 20, 30, 40, and 50 mg/L for PHB, PHT, LEV, LAC, ZON, and RUF; 2, 5, 10, 15, 20, 40, 60, 80, and 100 mg/L for FEL; 3, 7.5, 15, 22.5, 30, 60, 90, 120, and 150 mg/L for ETH; 0.5, 1, 2.5, 5, 7.5, 10, 20, 30, 40, and 50 mg/L for PRM, LTG, MHD, and CBZ; 0.2, 0.5, 1, 1.5, 2, 4, 6, 8, and 10 mg/L for DIOL and CBZ-E, and 0.1, 0.2, 0.5, 1, 1.5, 2, 4, 6, 8, and 10 mg/L for OXC. Twenty-five µL of the working solution was added to blank plasma samples to construct the calibration curves. Quality control (QC) samples were prepared separately from stock solutions at three concentration levels, representing low (QCL), middle (QCM), and high (QCH) points of the calibration curves (Table 1). All solutions were stored at 4 °C.

### 3.4. Sample Preparation

To 100 µL of plasma aliquots, 25 µL of calibration standard or QC standard solution containing IS was added. The samples were then vortexed for 30 s, and then 800 µL of ice-cold acetonitrile was added. After 30 s of vortexing, samples were centrifuged at 5000× *g* at 4 °C for 10 min. 800 µL of the organic phase was transferred and evaporated to dryness under a stream of nitrogen at 40 °C (Turbovap LV, Caliper, Hopkinton, MA, USA). To the dry residue, 100 μL of the methanol:water solution (50:50, *v*/*v*) was added and vortexed for 60 s. Samples were briefly centrifuged for 1 min at 16,100× *g*, and 80 µL were transferred to vials with inserts and submitted for analysis. Patients’ samples were prepared in the same manner by adding 25 µL of IS solution (50 mg/L) to 100 µL of patient plasma.

### 3.5. Method Validation

Validation was performed on three separate days according to US FDA guidelines for bioanalytical method validation [38].

#### 3.5.1. Selectivity

The method selectivity was evaluated by analyzing six blank plasma samples from different individuals to assess possible interference of matrix endogenous compounds at the retention times of the analytes and IS. Additionally, several drugs commonly co-administered with the analyzed AEDs were tested to check possible interferences at the retention time of each analyte.

#### 3.5.2. Linearity

The linearity of the method was assessed within the defined plasma concentration ranges by using calibration standards on three different days. Calibration curves were constructed by plotting the analyte–IS peak area ratio against the corresponding plasma concentration. The parameters of the calibration curves were calculated by non-weighted linear regression analysis. A high correlation coefficient (R^2^ > 0.99) was used as a criterion for linearity.

The lowest limit of quantification (LLOQ) was calculated based on the lowest concentration of each analyte that gives a coefficient of variation (CV) and bias value of ≤20%.

#### 3.5.3. Accuracy and Precision

The accuracy and precision of the method were assessed using five replicates of the QC samples covering low, medium, and high ranges of the calibration curve for each analyte. Intra-day accuracy and precision were assessed in a single run, while inter-day accuracy and precision were evaluated on three different days. The intra- and inter-day precision and accuracy of the method were determined as percent coefficient of variation (CV) and percent bias values, respectively. Obtained CV and bias should be within ±15%.

#### 3.5.4. Recovery

Recovery of the method was determined for each analyte and IS by measuring the peak area response of spiked plasma QC samples (QCL, QCM, and QCH) against the peak area response of aqueous QC samples at the same concentration (*n* = 3). Results are presented as mean recovery value ± SD considering all measurements.

#### 3.5.5. Stability

Stability was assessed at three concentration levels (QCL, QCM, and QCH) by comparing the data of the freshly prepared reference samples and stability samples (samples exposed to the test conditions for stability assessment). The freeze and thaw, short-term, long-term, stock solution, and autosampler stabilities of the analytes were investigated using three replicates of QC samples and determined by the percentage of calculated stability/reference samples ratio. The short-term stability of the analytes in plasma samples was assessed after 4 h storage at room temperature. Freeze and thaw stability was evaluated after three freeze and thaw cycles for aliquots stored at −80 °C for 24 h, thawed at room temperature, and refrozen for the next 24 h. The long-term stability was assessed by analyzing the QC plasma aliquots that had been stored at −80 °C for 45 days. The stability of stock solutions was investigated after storage at 4 °C for 7 days. Autosampler stability was evaluated by keeping prepared QC samples in the autosampler at 10 °C for 24 h and re-injecting them. A stability/reference samples ratio of 85–115% was set as an acceptance criterion.

## 4. Conclusions

A simple, selective, accurate, and precise HPLC-UV method for simultaneous determination of CBZ, PHB, PHT, PRM, FEL, ETH, OXC, ZON, RUF, LTG, LEV, and LAC, including three metabolites (CBZ-E, DIOL, and MHD) by using 100 µL of human plasma is described. The main advantage of our method is the simplicity of sample preparation and instrumentation, the appropriate analytical range for routine monitoring, and the suitability of the method for TDM of AEDs in sensitive populations such as pediatrics, pregnant women, elderly or critically ill patients, where small volumes of blood samples are recommended from the ethical viewpoint. The suitability of the presented method for routine TDM of AEDs was demonstrated by analysis of plasma samples from patients with epilepsy on combination AED therapy.

## Figures and Tables

**Table 1:** Calibration parameters and accuracy and precision values at the lower limit of quantification (LLOQ) values.

Analyte	Therapeutic Range (mg/L)	Analytical Range (mg/L)	Retention Time (min)	LLOQ (mg/L)	CV (%)	Bias (%)	Calibration Parameters			
							Equation	SD Slope	SD Intercept	R^2^
LEV	12–46	1–50	4.6	1.06 ± 0.12	11.93	6.30	Y = 0.0164x − 0.0027	0.0007	0.0036	0.9998
ETH	40–100	3–150	7.3	2.86 ± 0.46	17.54	−4.57	Y = 0.0033x + 0.0029	0.0001	0.0018	0.9993
ZON	10–40	1–50	9.7	0.94 ± 0.06	6.59	−5.59	Y = 0.0459x + 0.0063	0.0012	0.0054	0.9998
PRM	5–10	0.5–50	11.2	0.94 ± 0.03	3.98	−6.31	Y = 0.0321x + 0.0075	0.0057	0.0025	0.9998
LAC	10–20	1–50	11.7	0.97 ± 0.12	14.05	−2.86	Y = 0.0333x + 0.0148	0.0009	0.0038	0.9998
FEL	30–60	2–100	12.3	1.84 ± 0.12	7.21	−8.21	Y = 0.0254x + 0.0181	0.0002	0.0061	0.9997
RUF	30–40	1–50	12.7	0.96 ± 0.08	9.06	−4.49	Y = 0.0647x + 0.0197	0.0005	0.0087	0.9998
LTG	2.5–15	0.5–50	13.9	0.44 ± 0.04	10.12	−11.55	Y = 0.1213x + 0.0227	0.0024	0.0132	0.9998
DIOL	NE *	0.2–10	14.5	0.17 ± 0.01	9.25	−13.00	Y = 0.0846x + 0.0099	0.0022	0.0004	0.9994
PHB	10–40	1–50	15.2	0.97 ± 0.08	8.87	−3.32	Y = 0.0522x + 0.0072	0.0017	0.0041	0.9998
MHD	3–35	0.5–50	16.5	0.45 ± 0.04	10.71	−9.64	Y = 0.0859x + 0.0140	0.0045	0.0105	0.9998
CBZ-E	NE *	0.2–10	18.9	0.16 ± 0.01	4.96	−19.69	Y = 0.1173x + 0.0060	0.0033	0.0071	0.9996
OXC	NE *	0.1–10	19.8	0.11 ± 0.02	19.64	7.19	Y = 0.0619x−0.0003	0.0089	0.0063	0.9995
PHT	10–20	1–50	20.9	0.91 ± 0.08	9.49	−9.40	Y = 0.0594x + 0.0103	0.0019	0.0057	0.9997
CBZ	4–12	0.5–50	21.4	0.44 ± 0.03	7.71	−12.98	Y = 0.0915x + 0.0144	0.0008	0.0071	0.9997

* NE-not established.

**Table 2:** Inter- and intra-day accuracy (bias) and precision (CV) of the analytes in plasma samples at the low, middle, and high concentrations of the calibration ranges.

Analyte	C_nominal_ (mg/L)		Intra-Day (*n* = 5)			Inter-Day (*n* = 15)		
			C_measured_ (mg/L)	CV (%)	Bias (%)	C_measured_ (mg/L)	CV (%)	Bias (%)
	QC_L_	3	3.20 ± 0.02	0.74	6.76	3.03 ± 0.13	4.11	0.90
LEV	QC_M_	15	15.09 ± 0.02	0.21	0.58	15.30 ± 0.39	2.53	1.98
	QC_H_	45	45.54 ± 0.12	0.26	1.21	45.24 ± 0.28	0.62	0.54
	QC_L_	9	8.47 ± 0.13	1.48	−5.86	8.73 ± 0.35	4.00	−3.01
ETH	QC_M_	45	42.71 ± 0.12	0.27	−5.09	45.81 ± 2.70	5.09	1.79
	QC_H_	135	135.72 ± 0.55	0.41	0.53	136.65 ± 1.53	1.12	1.22
	QC_L_	3	3.07 ± 0.01	0.10	2.42	3.07 ± 0.09	2.95	2.44
ZON	QC_M_	15	15.15 ± 0.02	0.14	0.97	15.42 ± 0.45	2.91	2.81
	QC_H_	45	45.37 ± 0.13	0.28	0.82	44.99 ± 0.33	0.98	−0.02
	QC_L_	1.5	1.54 ± 0.01	0.7	2.72	1.53 ± 0.04	2.58	2.12
PRM	QC_M_	15	15.21 ± 0.02	0.14	1.40	15.41 ± 0.37	2.38	2.75
	QC_H_	45	45.44 ± 0.15	0.33	0.97	44.95 ± 0.44	0.98	−0.11
	QC_L_	3	3.01 ± 0.03	1.15	0.28	3.08 ± 0.08	2.57	2.57
LAC	QC_M_	15	15.14 ± 0.02	0.16	0.97	15.44 ± 0.47	3.01	2.90
	QC_H_	45	45.41 ± 0.13	0.29	0.92	45.02 ± 0.33	0.73	0.04
	QC_L_	6	6.14 ± 0.01	0.23	2.32	6.18 ± 0.24	3.92	2.97
FEL	QC_M_	30	30.41 ± 0.03	0.09	1.37	30.95 ± 0.86	2.77	3.16
	QC_H_	90	90.74 ± 0.14	0.16	0.82	89.97 ± 0.64	0.71	−0.03
	QC_L_	3	3.08 ± 0.02	0.61	2.71	3.09 ± 0.09	2.99	3.16
RUF	QC_M_	15	15.14 ± 0.03	0.18	0.96	15.43 ± 0.48	3.09	2.92
	QC_H_	45	45.38 ± 0.14	0.31	0.85	45.04 ± 0.29	0.64	0.12
	QC_L_	1.5	1.55 ± 0.005	0.32	2.95	1.54 ± 0.04	2.42	2.42
LTG	QC_M_	15	15.15 ± 0.02	0.16	1.02	15.43 ± 0.43	2.78	2.86
	QC_H_	45	45.46 ± 0.14	0.32	1.02	45.04 ± 0.36	0.80	0.09
	QC_L_	0.6	0.56 ± 0.04	7.64	−5.97	0.60 ± 0.02	3.22	−0.75
DIOL	QC_M_	3	3.00 ± 0.08	2.59	−0.09	3.07 ± 0.11	3.61	2.45
	QC_H_	9	9.08 ± 0.11	1.21	0.90	9.05 ± 0.06	0.65	0.54
	QC_L_	3	3.04 ± 0.01	0.46	1.17	3.54 ± 0.08	2.52	2.39
PHB	QC_M_	15	15.17 ± 0.05	0.34	1.12	15.46 ± 0.5	3.23	3.08
	QC_H_	45	45.34 ± 0.12	0.27	0.75	45.06 ± 0.25	0.55	0.13
	QC_L_	1.5	3.08 ± 0.005	0.17	2.67	3.07 ± 0.04	2.71	2.40
MHD	QC_M_	15	15.22 ± 0.02	0.16	1.44	15.46 ± 0.44	2.86	3.30
	QC_H_	45	45.44 ± 0.14	0.30	0.97	45.00 ± 0.38	0.85	−0.01
	QC_L_	0.6	0.58 ± 0.10	3.12	3.09	0.57 ± 0.023	4.09	−5.09
CBZ-E	QC_M_	3	2.98 ± 0.08	0.55	−0.02	3.03 ± 0.065	2.16	0.89
	QC_H_	9	9.12 ± 0.21	0.47	1.29	9.01 ± 0.090	1.00	0.13
	QC_L_	0.3	0.29 ± 0.16	10.57	0.98	0.30 ± 0.01	4.10	−0.63
OXC	QC_M_	3	2.83 ± 0.44	3.09	−5.67	2.94 ± 0.09	3.32	−2.11
	QC_H_	9	9.36 ± 1.98	4.23	3.96	9.05 ± 0.27	2.94	0.56
	QC_L_	3	3.07 ± 0.02	0.75	2.49	3.07 ± 0.09	2.81	2.35
PHT	QC_M_	15	15.19 ± 0.04	0.26	1.27	15.49 ± 0.48	3.11	3.30
	QC_H_	45	45.26 ± 0.15	0.32	0.58	44.95 ± 0.27	0.61	−0.12
	QC_L_	1.5	1.55 ± 0.01	0.87	3.08	1.54 ± 0.05	3.26	2.52
CBZ	QC_M_	15	15.15 ± 0.02	0.14	1.02	15.45 ± 0.48	3.09	2.99
	QC_H_	45	45.33 ± 0.15	0.34	0.74	44.92 ± 0.37	0.82	−0.18

**Table 3:** Recovery and stability data. Results are presented as mean value ± SD (%), taking into account all measurements.

Analyte	Recovery	Autosampler Stability	Stock Solution	Freeze and Thaw Stability	Short Term Stability	Long-Term Stability
LEV	96.2 ± 3.3	100.6 ± 2.1	102.3 ± 4.2	100.3 ± 0.9	101.3 ± 2.1	101.4 ± 1.8
ETH	93.4 ± 4.3	98.9 ± 1.1	101.6 ± 6.3	104.6 ± 3.9	105.7 ± 2.2	103.1 ± 4.9
ZON	99.7 ± 0.7	100.6 ± 0.4	101.4 ± 1.3	99.9 ± 0.2	101.0 ± 0.7	99.2 ± 6.3
PRM	99.8 ± 1.1	100.7 ± 0.5	101.7 ± 0.8	99.6 ± 0.3	100.9 ± 0.7	98.8 ± 6.6
LAC	101.4 ± 1.0	100.4 ± 0.5	100.2 ± 0.5	100.1 ± 0.4	100.9 ±0.8	98.7 ± 5.1
FEL	100.9 ± 0.7	100.8 ± 0.4	101.3 ± 1.3	99.1 ± 0.9	100.9 ± 0.7	98.1 ± 5.3
RUF	100.4 ± 0.4	100.1 ± 0.8	101.6 ± 2.1	100.2 ± 0.5	101.0 ± 0.8	100.2 ± 6.5
LTG	100.2 ± 0.2	100.4 ± 0.4	100.5 ± 1.8	99.3 ± 0.5	100.9 ± 0.7	98.0 ± 5.8
DIOL	102.2 ± 1.8	101.8 ± 2.4	102.1 ± 4.2	102.8 ± 3.4	102.1 ± 2.7	99.0 ± 4.9
PHB	100.4 ± 0.5	101.0 ± 1.1	100.8 ± 1.3	99.4 ± 0.6	100.4 ± 1.0	98.9 ± 6.0
MHD	99.8 ± 0.4	100.2 ± 0.4	101.5 ± 1.3	99.9 ± 0.02	100.6 ± 0.7	99.0 ± 6.1
CBZ-E	99.6 ± 0.6	100.2 ± 1.0	100.2 ± 1.3	101.7 ± 2.7	101.8 ± 0.9	101.8 ± 7.2
OXC	83.9 ± 5.2	91.4 ± 3.5	95.5 ± 2.5	95.3 ± 1.6	98.5 ± 4.3	101.6 ± 6.2
PHT	99.7 ± 0.8	101.9 ± 1.7	102.3 ± 2.2	100.8 ± 1.7	101.3 ± 0.4	99.4 ± 7.1
CBZ	99.6 ± 1.4	100.8 ± 0.9	101.6 ± 1.4	99.6 ± 0.5	100.7 ± 0.6	99.5 ± 6.9
IS	100.2 ± 1.6	100.8 ± 3.3	103.1 ± 1.3	97.8 ± 1.4	99.5 ± 4.0	98.0 ± 0.6

**Table 4:** Measured concentrations of the AEDs and their metabolites (MHD, DIOL, and CBZ-E) in plasma samples from patients with epilepsy.

ID	Therapy (mg/day)	Measured Concentration (mg/L)											
		LTG	LEV	OXC	MHD	DIOL	CBZ	CBZ-E	ETH	PHB	PHT	LAC	ZON
1	OXC (450) LEV (1050) ETH (475)	/	25	0.4	12	2.7	/	/	52	/	/	/	/
2	OXC (450) Vigabatrin (500)	/	/	0.3	8.7	1.2	/	/	/	/	/	/	/
3	OXC (450) Topiramate (200)	/	/	1.7	14	1.5	/	/	/	/	/	/	/
4	LTG(400) OXC (1200) PHB (50)	4.6	/	0.3	11	0.6	/	/	/	2.2	/	/	/
5	LTG (400) OXC (1200)	2.2	/	0.4	8.8	0.2	/	/	/	/	/	/	/
6	LTG (600) PHT (300) Pregabaline (150)	4.2	/	/	/	/	/	/	/	/	1.2	/	/
7	LTG (400) PHT (100)	2.8	/	/	/	/	/	/	/	/	1.1	/	/
8	LTG (150) ZON (300) Valproic acid (750)	5.6	/	/	/	/	/	/	/	/	/	/	8.3
9	LTG (400) LAC (200)	5.9	/	/	/	/	/	/	/	/	/	4.4	/
10	LTG (300) CBZ(1200)	2.1	/	/	/	3.9	8.0	1.1	/	/	/	/	/
11	LTG (300) LEV (3500) Clonazepam (1)	2.8	39.7	/	/	/	/	/	/	/	/	/	/
12	LTG (250) PHB (250)	0.9	/	/	/	/	/	/	/	7.6	/	/	/
13	LTG (300) CBZ (800)	1.4	/	/	/	0.7	3.9	0.3	/	/	/	/	/

**Table 5:** HPLC gradient elution.

Time (min)	% KH_2_PO_4_ (25 mM; pH 5.1)	% Methanol
0	75	25
5	70	30
10	60	40
15	58	42
19	30	70
23	30	70
23.1	75	25
27	75	25

## Data Availability

The data presented in this study are available on request from the authors.
